# Electrocatalytic Oxidation of Benzaldehyde on Gold Nanoparticles Supported on Titanium Dioxide

**DOI:** 10.3390/nano14121005

**Published:** 2024-06-10

**Authors:** Li Gong, Yu Jin, Shiling Zhao, Kaizhi Wang, Paulina R. Martínez-Alanis, Andreu Cabot

**Affiliations:** 1 Catalonia Institute for Energy Research–IREC Sant Adrià de Besòs, 08930 Barcelona, Spain; 2 Faculty of Chemistry, University of Barcelona, 08028 Barcelona, Spain; 3 Key Laboratory of Applied Organic Chemistry (SKLAOC), The Key Laboratory of Catalytic Engineering of Gansu Province, College of Chemistry and Chemical Engineering, Lanzhou University, Lanzhou 730000, China; jiny21@lzu.edu.cn; 4 School of Petrochemical Engineering, Lanzhou University of Technology, Lanzhou 730050, China; slzhao@lut.edu.cn; 5 Department of Chemistry, Shanghai Key Laboratory of Molecular Catalysis and Innovative Materials, Fudan University, Shanghai 200433, China; 6 ICREA, Pg. Lluís Companys 23, 08010 Barcelona, Spain

**Keywords:** electrooxidization, benzaldehyde, titanium dioxide, oxygen vacancy, Lewis acid active sites

## Abstract

The electrooxidation of organic compounds offers a promising strategy for producing value-added chemicals through environmentally sustainable processes. A key challenge in this field is the development of electrocatalysts that are both effective and durable. In this study, we grow gold nanoparticles (Au NPs) on the surface of various phases of titanium dioxide (TiO_2_) as highly effective electrooxidation catalysts. Subsequently, the samples are tested for the oxidation of benzaldehyde (BZH) to benzoic acid (BZA) coupled with a hydrogen evolution reaction (HER). We observe the support containing a combination of rutile and anatase phases to provide the highest activity. The excellent electrooxidation performance of this Au-TiO_2_ sample is correlated with its mixed-phase composition, large surface area, high oxygen vacancy content, and the presence of Lewis acid active sites on its surface. This catalyst demonstrates an overpotential of 0.467 V at 10 mA cm^−2^ in a 1 M KOH solution containing 20 mM BZH, and 0.387 V in 100 mM BZH, well below the oxygen evolution reaction (OER) overpotential. The electrooxidation of BZH not only serves as OER alternative in applications such as electrochemical hydrogen evolution, enhancing energy efficiency, but simultaneously allows for the generation of high-value byproducts such as BZA.

## 1. Introduction

The excessive dependence on fossil fuels exacerbates environmental pollution and contributes to geopolitical energy issues [1]. Integrating renewable energy sources such as solar, wind, and tidal power into the electricity grid can significantly reduce carbon emissions and contribute to a more uniform distribution of power. This integration also holds promise for supporting the burgeoning global clean hydrogen energy sector [2]. However, the realization of this vision hinges on the development of cost-effective energy storage technologies and particularly electrochemical technologies and materials for converting electrical energy into chemical energy [3].

One of the key processes in this field is the electrolysis of water, where the hydrogen evolution reaction (HER) at the cathode is highly regarded due to its high energy density and clean attributes [4,5]. Conversely, the oxygen evolution reaction (OER) at the anode, with a substantial thermodynamic potential (1.23 V vs. RHE), impedes multiple proton–electron transfer kinetics and consumes over 80% of the electricity used in water electrolysis. Additionally, the OER’s product, oxygen, has relatively low economic value. Given these factors, substituting the OER with the electrooxidation of some biomass-derivate that have more favorable thermodynamics offers a strategic avenue for more energy-efficient production while also enabling the simultaneous creation of high-value-added biomass-derived chemicals [6,7,8]. Organic electrochemical oxidation reactions, including alcohol and aldehyde oxidations [9,10], typically require much lower theoretical thermodynamic potentials than the OER, as evidenced by reactions like the urea oxidation reaction (0.37 V vs. RHE) [11], hydrazine oxidation reaction (−0.33 V vs. RHE) [12], ammonia oxidation reaction (0.06 V vs. RHE) [13,14], and glucose oxidation reaction (0.05 V vs. RHE) [15]. These reactions not only promise greater energy efficiency than OER-based water splitting but also expand the potential applications of electrolytic processes in producing economically significant chemicals derived from biomass products, i.e., having net-zero and even negative CO_2_ emissions.

Numerous catalysts based on Co, Fe, Ni, and Cu-based materials dispersed on diverse high surface area supports such as carbon cloth (CC) or nickel foam (NF) have been employed as electrooxidation catalysts [8,11,15]. For example, Sun’s group has explored Ni_2_P/Ni/NF as electrocatalyst for the oxidation of furfural to 2-furoic acid coupled with H_2_ evolution [16]. Furthermore, Wang’s group reported a bipolar hydrogen production system, which couples the low-potential anodic oxidation of biomass aldehydes with cathodic HER. In this system, a two-electrode electrolyser for bipolar hydrogen production was assembled using Cu foam as the anode and Pt/C as the cathode, with serpentine flow channels. Under an applied voltage of only ~0.1 V, both the cathode and anode can simultaneously generate H_2_ [17]. We have also demonstrated the viability of the oxidation of other small molecules such as ethanol, methanol, urea, formate, and ethylene glycol [18,19].

As an electrocatalyst, gold (Au) is an excellent candidate for the oxidation of alcohols and aldehydes due to the facile formation of highly active Au-O-H intermediate states with reactants [20]. Au is generally dispersed onto substrates able to modulate its properties and minimize aggregation due to its high cost and relatively low abundance. Rutile and anatase titanium dioxide (TiO_2_) nanoparticles (NPs) with a bandgap of 3.1–3.2 eV are widely used in organic compound degradation and catalysis for their low cost, excellent stability, significantly large surface area, and adjustable electronic properties. The rutile phase exhibits higher catalytic activity, whereas the anatase structure is more stable [21,22]. TiO_2_-P25 belongs to the mixed-phase type, with an approximate weight ratio of 80/20 between anatase and rutile. The mixing of the two phases increases the oxygen defect density within the TiO_2_ lattice, enhancing the concentration of charge carriers. This leads to an increase in the number of holes, endowing it with a stronger ability to capture components, including water, oxygen, and organic compounds, on the surface.

To explore the performance of Au nanoparticles (NPs) loaded on TiO_2_ support as a potential electrooxidation catalyst, as well as the alternative electrocatalytic anodic reaction of the OER, here, we choose the benzaldehyde (BZH) electrooxidation to benzoic acid (BZA) as an OER alternative. BZH is widely acknowledged as a versatile platform for biomass intermediates, given its capability to be transformed into numerous highly valuable chemical feedstocks. The reduction products benzyl alcohol (BA) and hydrobenzoin (HDB), and the hydrolysis product toluene are important high-value-added chemicals [23,24]. Additionally, the BZH oxidation product BZA plays a crucial role as a precursor in the biosynthesis of numerous secondary metabolites and its salts are widely employed as food preservatives due to their antimicrobial properties [25]. Moreover, BZA serves as a key building block in the industrial synthesis of various organic compounds [26]. As the catalyst, we explore the use of Au NPs supported on three types of TiO_2_, anatase TiO_2_ (TiO_2_-A), rutile TiO_2_ (TiO_2_-R), and TiO_2_-P25 with a phase mixture. The catalyst’s physicochemical properties are thoroughly investigated and their performance toward the BZH electrooxidation to BZA is tested as a function of the support phase.

## 2. Materials and Methods

### 2.1. Reagents and Chemicals

All reagents and chemicals were used as received without any further purification. Metal oxides, TiO_2_-P25, anatase TiO_2_ (TiO_2_-A), and rutile TiO_2_ (TiO_2_-R) were supplied by Evonik. HAuCl_4_ꞏ4 H_2_O, sodium hydroxide (NaOH, 97%), and hydrochloric acid (HCl, 37%) were purchased from Sigma Aldrich.

### 2.2. Synthesis of the Au-TiO_2_-P25, Au- TiO_2_-A, and Au-TiO_2_-R

Au-loaded TiO_2_ samples were prepared by a modified impregnation method [27]. Briefly, 0.1 g powder TiO_2_ was uniformly dispersed in 10 mL of a 0.1 mg_Au_ mL^−1^ aqueous HAuCl_4_ solution (pH adjusted to 9 with 0.2 M NaOH). Following that, the mixture was heated at 80 °C for 2 h while vigorously stirred. Upon cooling to 25 °C, the suspension underwent filtration, followed by a comprehensive wash with deionized water. The material was subsequently dried in a vacuum oven at 60 °C for 12 h. Next, it underwent annealing in a 5% hydrogen/argon atmosphere (flow rate: 80 mL/min) at 350 °C for reduction with a duration of 4 h.

### 2.3. Characterizations

Powder X-ray diffraction (XRD) patterns were collected at 40 kV and 40 mA with Cu Kα radiation (λ = 1.5406 Å). X-ray photoelectron spectroscopy (XPS) was conducted on a ThermoFisher (ESCALAB 250Xi) system with Mono Al Kα radiation. The vacuum of the analysis chamber was approximately 2 × 10^−9^ mbar. The energy, voltage, and beam current were 1486.6 eV, 16 kV, and 15 mA, respectively. Accurate binding energies (±0.1 eV) were corrected with respect to the position of the adventitious C 1s peak at 284.6 eV. To determine the specific acid sites of Brønsted or Lewis acid sites, Fourier-transform infrared spectroscopy (FTIR) spectra of pyridine adsorption were collected on a Thermo-Fisher Nicolet iS50 spectrometer. Self-supporting pellets made of the catalysts were placed in a flow cell and evacuated under reduced pressure at 300 °C for 2 h to remove the adsorbed moisture from the material surface. Spectrum was recorded in the range of 4000–650 cm^−1^ at 150 °C. Transmission electron microscopy (TEM) measurements were carried out on a Tecnai G_2_ F20 S-Twin electron microscope, operating at 200 kV. H_2_–temperature programmed reduction (H_2_-TPR) profiles were conducted on the Autochem HP system (Micromeritics Instrument Corp.). The particle size distribution was determined using the software Nano Measurer developed by the Department of Chemistry at Fudan University. At least 50 particles were manually marked and measured from each sample. The chemical compositions of catalysts were analyzed on a PerkinElmer inductively coupled plasma optical emission spectrometer (ICP-OES) Optima 8300 DV.

### 2.4. Product Identification and Conversion and Selectivity Quantification

Organic compounds were analyzed via high-performance liquid chromatography (HPLC) using an Agilent 1200 series apparatus operating at 25 °C. The HPLC setup comprised an ultraviolet–visible detector and a 4.6 mm × 150 mm Shim-pack GWS 5 μm C18 column. Elution employed solvents A (5 mM ammonium formate aqueous solution) and B (acetonitrile), with a gradient program of 60% B and 40% A over 11 min. The flow rate was maintained at 0.5 mL/s, and the injection volume was 1 μL. To exclude the effect of the BZH Cannizzaro reaction on the product quantification, the samples need to be injected into the HPLC system as soon as possible. Calibration curves of standard chemicals facilitated product determination and quantification. Total conversion was computed using the following formula:

(1) $$\mathrm{Conv}.\;\%=\frac{moles\;of\;BZH\;consumed}{initial\;moles\;of\;BZH}\times 100\%$$

### 2.5. Electrochemistry Tests

The electrochemical performance of the electrocatalyst was evaluated on a CHI66E (Shanghai Chenhua) workstation using a 3-electrode system in an H-cell reactor divided by a Nafion 117 proton exchange membrane. The working electrode was prepared as follows: 6 mg powder was dispersed into 500 μL H_2_O, 420 μL ethanol, and 80 μL 5% Nafion solution. For glassy carbon working electrode preparation, 8 μL ink solution was dropped on a glassy carbon working electrode (d = 0.15 cm, ~0.7 mg cm^−2^) and left to dry. Carbon cloth (4 cm^2^) and Ag/AgCl (KCl-saturated) were used as counter and reference electrodes, respectively. Linear sweep voltammetry (LSV) was tested with a glassy carbon working electrode and results were calibrated with the Nernst Equation (2) to the reference hydrogen electrode (RHE) without iR correction. Electrochemical impedance spectroscopy (EIS) was tested at 1.6 V vs. RHE. Dropping the ink to 1 × 2 cm^2^ carbon cloth (keep loading mass: ~1.5 mg cm^2^) as working electrode for conversion and stability tests.

(2) $$E_{RHE}=E+0.0591\times pH+E_{Ag/AgCl}^{\theta }$$

## 3. Results and Discussion

**Scheme 1:**
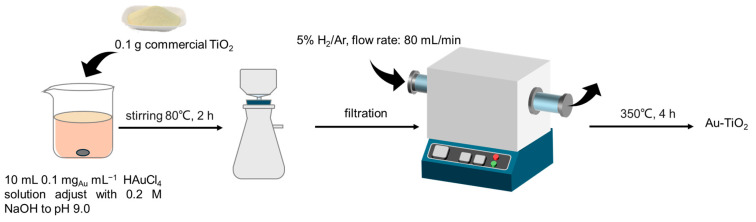
Scheme of the preparation process of the electrocatalyst Au-TiO_2_.

**Figure 1:**
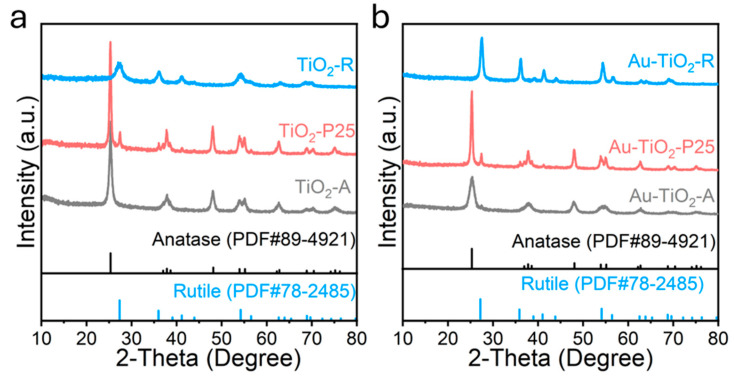
XRD patterns of the different catalytic materials, (**a**) TiO_2_, (**b**) Au-TiO_2_, including reference patterns for rutile and anatase.

The three catalytic materials, Au-TiO_2_-P25, Au-TiO_2_-A, and Au-TiO_2_-R, were prepared by impregnation followed by H_2_ reduction process (Scheme 1, see details in the Section 2). XRD patterns are displayed in Figure 1a. The peaks at 25.35°, 37.01°, 37.85°, 38.64°, 48.14°, 53.97°, and 55.18° correspond to anatase TiO_2_ (TiO_2_ PDF#89-4921), while peaks at 27.44°, 36.08°, 41.24°, 54.32°, 56.62°, and 69.00° are assigned to rutile TiO_2_ (TiO_2_ PDF#78-2485). Notice how the peaks co-exist in the mixed phase TiO_2_-P25. The XRD patterns of the materials after the Au loading (Figure 1b) reveal that the crystal structure of TiO_2_ is maintained. The Au peaks are not prominently observed, potentially due to the low loading.

**Figure 2:**
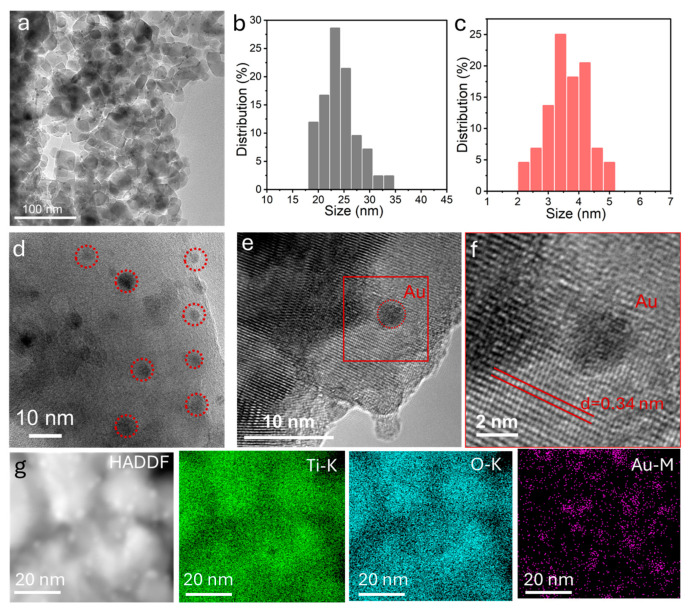
(**a**) TEM image of Au-TiO_2_-P25. (**b**) TiO_2_ particle size histogram of Au-TiO_2_-P25. (**c**) TEM image of Au-TiO_2_-P25. (**d**) Au particle size histogram of Au-TiO_2_-P25. (**e**) HRTEM image of Au-TiO_2_-P25. (**f**) Magnified red framed area of an Au NP and TiO_2_ lattice space of 0.34 nm. (**g**) STEM-EDS chemical composition maps of Au-TiO_2_-P25.

Figure 2a shows the regular polyhedral morphologies of TiO_2_-P25 NPs with a uniform distribution of Au NPs. We utilized particle analysis software to perform particle size measurements on the TiO_2_ NPs and Au NPs as displayed in Figure 2b,c, respectively. TiO_2_-P25 NPs had an aveage size of 24 nm (Figure 2b). Numerous Au NPs with a size of around 3.5 nm are distributed on their surface (Figure 2c,d). Both other materials, Au-TiO_2_-A and Au-TiO_2_-R, show similar morphologies and Au particle sizes (Figure S1). HRTEM images in Figure 2e confirm both the crystallographic structure of TiO_2_ in Au-TiO_2_-P25 and the presence of crystalline Au NPs. From the STEM-EDS chemical composition maps of Au-TiO_2_-P25 (Figure 2g), it can be observed that Ti and O are uniformly distributed throughout the entire material, as well as the uniform distribution of Au particles. The amount of Au was determined by ICP and found to match the nominal 1 wt% concentration introduced.

**Figure 3:**
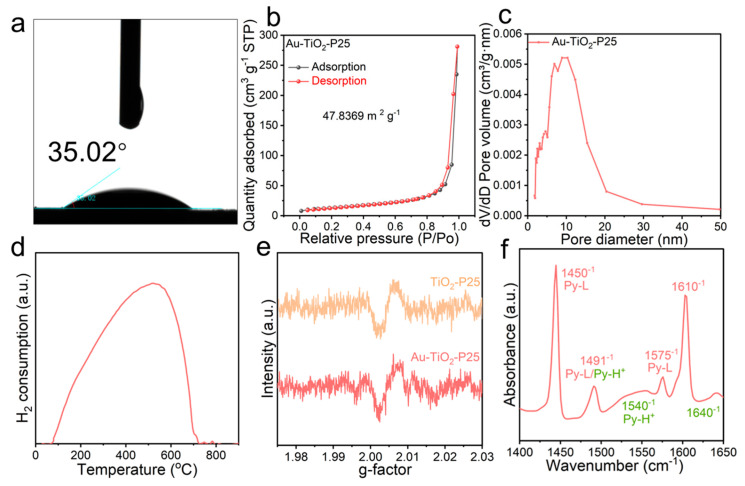
(**a**) A drop of water on Au-TiO_2_-P25; (**b**) nitrogen adsorption–desorption isothermal curves and (**c**) pore diameter distribution of Au-TiO_2_-P25; (**d**) H_2_–temperature programmed reduction (H_2_-TPR) profiles of Au-TiO_2_-P25; (**e**) EPR signals of TiO_2_-P25 and Au-TiO_2_-P25; (**f**) FTIR-Py spectrum tested at 150 °C to unravel the Brønsted/Lewis acid sites on the surface of Au-TiO_2_-P25.

**Figure 4:**
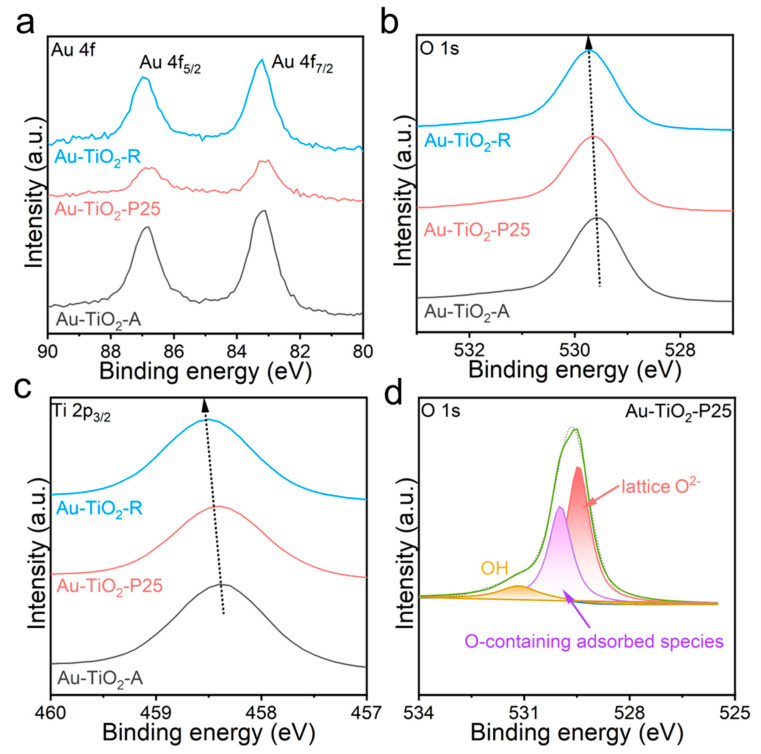
XPS spectra of (**a**) Au 4f; (**b**) Ti 2p_3/2_; (**c**,**d**) O 1s in materials Au-TiO_2_-P25, Au-TiO_2_-A; and Au-TiO_2_-R.

Au-TiO_2_ samples show a hydrophilic surface with a static water contact angle of about 35° (Figure 3a). N_2_ adsorption–desorption isotherms of the four materials tested, TiO_2_-P25, Au-TiO_2_-P25, Au-TiO_2_-R, and Au-TiO_2_-A, show type IV with H1 hysteresis loops characteristic of mesoporous structures with a Brunauer–Emmett–Teller (BET) surface area of 49 m^2^ g^−1^, 48 m^2^ g^−1^, 31 m^2^ g^−1^, and 41 m^2^ g^−1^, respectively (Figure 4b and Figure S2a,c,e). Au-TiO_2_-P25 has a significant distribution of mesopores with pore sizes around 12 nm. H_2_-TPR curves (Figure 3d) show one broad peak from 80 °C to 700 °C, which is assigned to the reduction of bulk TiO_2_ species. The electron spin resonance (EPR) spectra of TiO_2_-P25 and Au-TiO_2_-P25 both show noticeable signals at g = 2.004 (Figure 3e) ascribed to the existence of oxygen vacancies. The very similar spectra obtained indicate that the amount of surface oxygen vacancies is independent of the Au NP modification.

The interaction of Brønsted/Lewis acid sites with alkaline probe molecules on the surface of Au-TiO_2_-P25 was investigated using pyridine-adsorbed FTIR. As shown in Figure S3, at different active sites on the material surface, pyridine exhibits varied adsorption behaviors. The infrared characteristic peaks and intensities of the probe molecule pyridine can be used to discern the types of active sites on the material surface. Brønsted acid sites and Lewis acid sites on the catalyst surface are identified at 1540 cm^−1^ and 1450 cm^−1^, respectively. The signal at 1610 cm^−1^ was also ascribed to the strong Lewis-bound peak pyridine of pyridine ring vibration. The peak at 1640 cm^−1^ was ascribed to the pyridinium ion ring vibration owing to pyridine molecules bound to Brønsted acid sites. The in-plane ring vibration absorption peak of pyridine molecules was observed at 1575 cm^−1^. The C-H vibration absorption peak on the pyridine molecule was observed at 1491 cm^−1^ [28]. From the FTIR spectrum, we conclude that there is a notable density of Lewis acid sites on the catalyst surface but barely Brønsted sites. The electron-rich O in carbonyl groups tends to adsorb onto Lewis acidic sites for its electron-accepting property; therefore, accelerating the reaction rate. Several previous works have proved the efficiency of Lewis acid sites for electrooxidization reactions such as OER [29,30] and alcohol electrooxidization [31,32].

Figure 4a displays the Au 4f XPS spectra of the different materials. Peaks at 83.20 eV and 86.85 eV are assigned to Au 4f_7/2_ and Au 4f_5/2_ electronic states of metallic Au. The Ti 2p_3/2_ XPS spectrum of Au-TiO_2_-P25 shows one peak at 458.36 eV. This peak is redshifted for Au-TiO_2_-A and blueshifted for Au-TiO_2_-R. The O 1s XPS spectrum exhibits similar binding energy shifts (Figure 4c). The simultaneous shift in the Ti 2p and O 1s spectra is associated with a shift in the Fermi level in the TiO_2_. Notice this shift is not followed by the Au spectra, denoting a relatively independent electronic structure, consistent with our previous observations on SnO_2_ [33]. The O 1s XPS spectra can be deconvoluted in three peaks at 529.41 eV, 529.92 eV, and 531.1 eV corresponding to lattice O^2−^, O-containing adsorbed species, and OH groups on the material surface.

**Figure 5:**
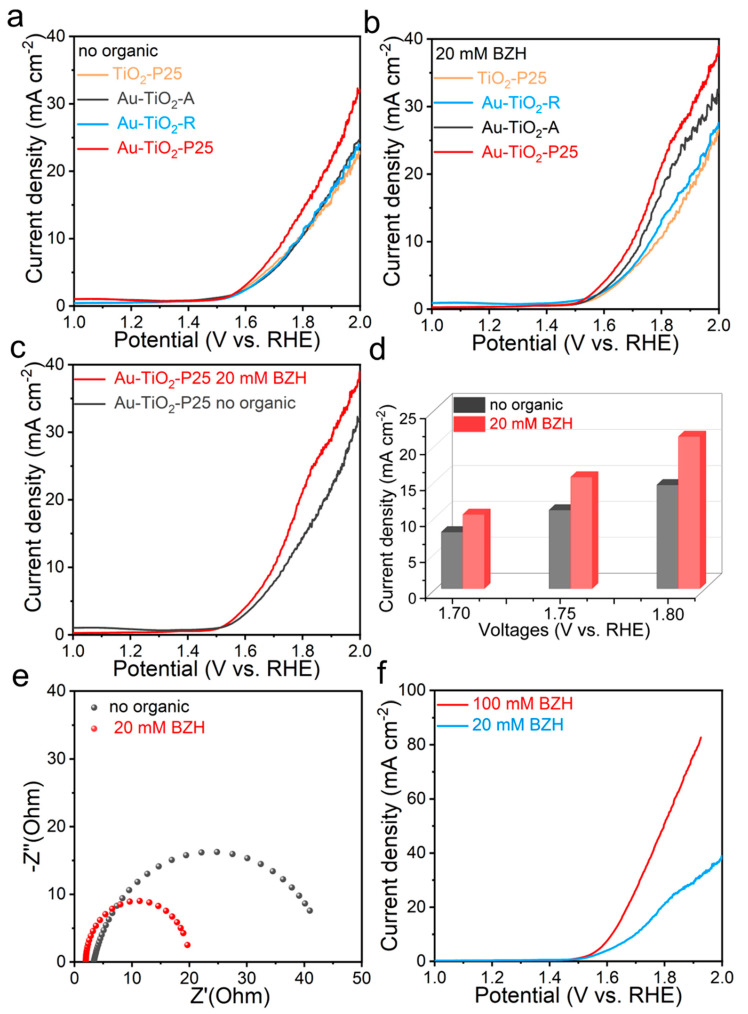
(**a**,**b**) LSV curves of different materials at a scan rate of 50 mV s^−1^ in (**a**) 1 M KOH solution; (**b**) 1 M KOH solution and 20 mM BZH. (**c**) Comparison of LSV curves of Au-TiO_2_-P25 in 1.0 M KOH and in the presence of 20 mM BZH. (**d**) Comparison of the current density of Au-TiO_2_-P25 at 1.70, 1.75, and 1.80 V vs. RHE in 1.0 M KOH and in addition of 20 mM BZH. (**e**) Nyquist plots of Au-TiO_2_-P25 electrode in 1.0 M KOH and in addition of 20 mM BZH. (**f**) Comparison of LSV curves when adding 20 mM BZH or 100 mM BZH.

The catalysts’ electrooxidation activity towards the OER was tested using an H-cell reactor with a three-electrode system. Au-TiO_2_ was supported on a glassy carbon as the working electrode, Ag/AgCl (KCl-saturated) as the reference electrode, and a 4 cm^2^ carbon cloth was used as the counter electrode. The electrooxidation performance was tested in 1 M KOH solution by LSV at a scan rate of 50 mV s^−1^, and the results as shown in Figure 5a. Au-TiO_2_-P25 showed the highest activity towards OER, with an overpotential of 0.505 V at 10 mA cm^−2^. When adding BZH into the system, the overpotentials were significantly decreased for all materials (Figure 5b). Still, Au-TiO_2_-P25 showed the lowest overpotential of 0.467 V at 10 mA cm^−2^ (Figure 5c). Moreover, the current densities at 1.70, 1.75, and 1.80 V vs. RHE in the presence of BZH were all larger than those in the absence of BZH (Figure 5d).

The Nyquist plot of the EIS spectra of Au-TiO_2_-P25 is shown in Figure 5e. Upon adding BZH, the semicircle diameter shrinks from 41 Ω to 18 Ω. The solution resistance also decreased from approximately 4 Ω to 2 Ω. When increasing the concentration of BZH to 100 mM, the overpotential further decreased to 0.387 V at 10 mA cm^−2^. The above data demonstrate both the excellent performance of catalyst Au-TiO_2_-P25 in the electrooxidation reaction and that the addition of BZH to the system can reduce the energy consumption of the redox electrochemical reaction that produces hydrogen at the cathode, the carbon cloth counter electrode.

**Figure 6:**
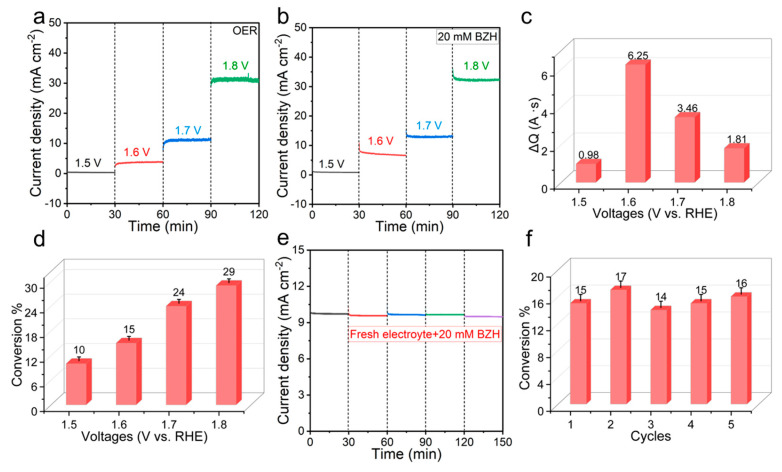
(**a**) ORE i-t curve in 1 M KOH at different voltages vs. RHE. (**b**) OER + BZH electrooxidation i-t curves in 1 M KOH with 20 mM BZH at different voltages vs. RHE. (**c**) Accumulated charge difference (DQ) between BZH electrooxidation and OER within 30 min at different voltages. (**d**) Conversion of BZH to BZA within 30 min as a function of the applied voltage. (**e**) Stability tests of 5 cycles i-t curves at constant voltage 1.6 V vs. RHE. (**f**) Conversion of BZH to BZA during 5 consecutive 30-min periods.

To further evaluate the catalyst performance, the electrocatalyst Au-TiO_2_-P25 was supported onto carbon cloth and its efficiency was assessed towards OER and BZH electrooxidation reactions. The tests were conducted using an immersion area of 1 × 1 cm^2^ in 1 M KOH electrolyte in the absence or the presence of BZH, and at various voltages within a 30-min timeframe. Fresh electrode materials were used for each experiment. Figure 6a illustrates the sustained OER performance of the electrocatalyst Au-TiO_2_-P25 over 30 min at voltages of 1.5 V, 1.6 V, 1.7 V, and 1.8 V vs. RHE. The current density increased with the voltage. Upon subsequent addition of 20 mM BZH to the system, the current density further increased (Figure 6b). The change in current density, associated with the BZH electrooxidation, was determined by integrating the charge (Q) passing through the system at each voltage during BZH electrooxidation, and then subtracting the corresponding OER values (Figure 6c). The difference in Q values (DQ) shows no monotonous pattern, but it increased with voltage in the range from 1.5 V to 1.6 V and decreased with voltage at higher applied potentials. This is related to the increase in OER at voltages above 1.6 V. Still, the conversion of BZH to BZA increased with the voltage from 10% at 1.6 V vs. RHE to 29% at 1.8 V vs. RHE (Figure 6d). To assess the electrocatalyst stability, we conducted five consecutive 30-min cyclic tests. From Figure 6e, it can be observed that the catalyst exhibits excellent stability with no major variation in current density at constant voltages 1.6 V vs. RHE of five cycles with 30 min/ cycle. Furthermore, the conversion rate of BZH remained constant at 15–16% (Figure 6f).

**Figure 7:**
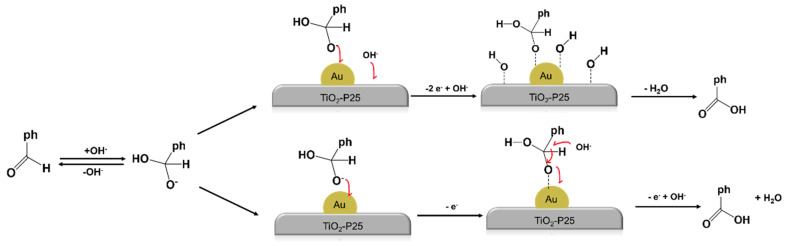
Illustration of electrooxidization pathways of BZH on Au-TiO_2_-P25. Arrows represent the direction of paired electron transfer, while half-arrows represent single-electron transfer.

Under strong alkaline conditions, BZH undergoes the autoxidation–reduction reaction known as the Cannizzaro reaction. The pre-step of the BZH electrooxidation reaction shares with the Cannizzaro reaction the first rapid and crucial step of formation of an intermediate by combining BZH with hydroxide ions, as shown in Figure 7. The high OH^−^ concentration at pH 14 strongly promotes this step (Figure S4). Following this, when applying positive voltages, there are two possible reaction pathways, both involving the intermediate product bound to gold with the oxygen atom. A possible mechanism is that both electrons transfer stepwise through the solution to Au (downside). Another possibility is that the two electrons transfer separately. One electron moves from the oxygen of the intermediate to the electrode material, while the other electron transfers from the hydroxide ion to the electrode material, forming a hydroxyl adsorption state. Subsequently, the C-H bond breaks, combining with hydroxyl to form a water molecule that is then removed [34,35,36]. Since TiO_2_ itself also exhibits electrocatalytic properties towards OER and electrooxidization of BZH, the activity of the material originates from the combined effect of Au and the substrate TiO_2_. Therefore, we are more inclined towards a mechanism where the two electrons transfer separately. In this scenario, oxygen vacancies and Lewis basic sites effectively accept electrons transferred from hydroxide ions to form adsorbed hydroxyl groups, increasing the probability of bonding with H on intermediates, thus enhancing reaction efficiency. Due to the consistent loading amount of Au (~1%), the outstanding performance of Au-TiO_2_-P25 stems from the strong adsorption capability of TiO_2_ towards substrates, its Lewis acid sites properties and the mixed anatase/rutile phase of TiO_2_ catalysts with increased surface area and enhanced defect density.

## 4. Conclusions

This study demonstrates the electrooxidation of BZH into BZA in an alkaline 1.0 M KOH aqueous solution over different phase TiO_2_-based catalysts loaded with Au NPs. Among the different phases of TiO_2_ as support catalysts, a mixture of anatase and rutile with 1 wt% Au loading showed the best OER performance due to its unique properties of mixed-phase, larger surface area, sufficient surface oxygen defects and Lewis acid sites. A decrease in overpotential at a constant current density of 10 mA cm^−2^ was observed from 0.505 V to 0.476 V after the addition of 20 mM BZH into the anode chamber of the three-electrode H-cell test system. Finally, considering further energy-saving purposes, a higher concentration of 100 mM of BZH decreased the overpotential to 0.387 V consecutively. In addition, the catalyst demonstrated excellent stability.

## Data Availability

Data available on request from the authors.

## Supplementary Materials

The following supporting information can be downloaded at https://www.mdpi.com/article/10.3390/nano14121005/s1. Figure S1: (a,b) TEM image of Au-TiO_2_-R; (c,d) TEM image of Au-TiO_2_-A; Figure S2: Nitrogen adsorption-desorption isothermal curves of materials and their pore sizes distributions (a,b) TiO_2_-P25; (c,d) Au-TiO_2_-R; (e,f) Au-TiO_2_-A; Figure S3: Diagram of pyridine adsorption on Brønsted acid sites and Lewis acid sites; Figure S4: LSV curves of material Au-TiO_2_-P25 at a scan rate of 50 mV s^−1^ in 1 M KOH (pH 14) and 0.1 M KOH (pH 13).
