# Supplementary material for: Electrocatalytic Oxidation of Benzaldehyde on Gold Nanoparticles Supported on Titanium Dioxide
Source: Nanomaterials (Basel). 2024 Jun 10;14(12):1005. doi: 10.3390/nano14121005 (PMC11206298; doi:10.3390/nano14121005)
Supplement: Supplementary file 1 [file nanomaterials-14-01005-s001.zip › nanomaterials-3034885-SI.pdf]

Supplementary Materials

Electrocatalytic Oxidation of Benzaldehyde on Gold Nanoparticles Supported on Titanium Dioxide

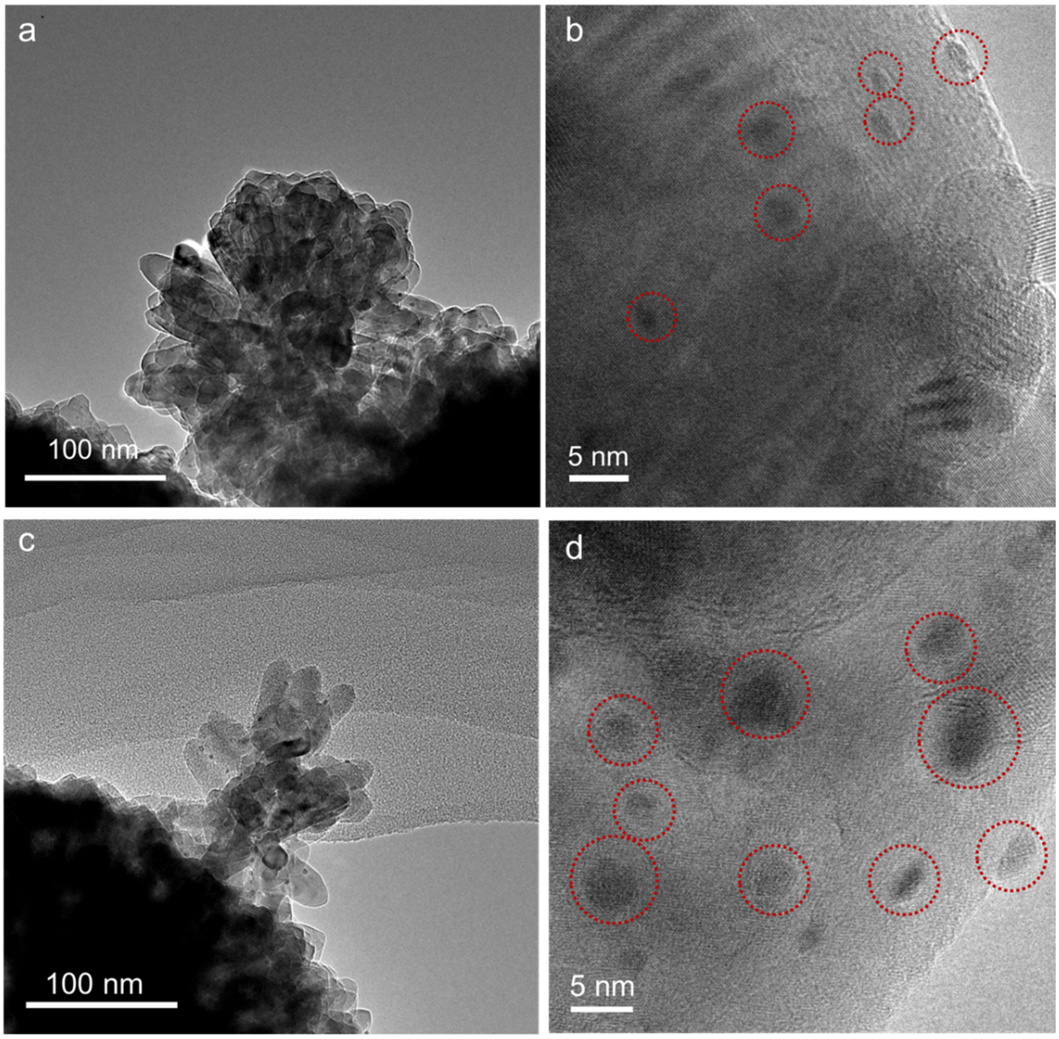

Figure S1. (a-b) TEM image of Au-TiO_2-R; (c-d) TEM image of Au-TiO_2-A.

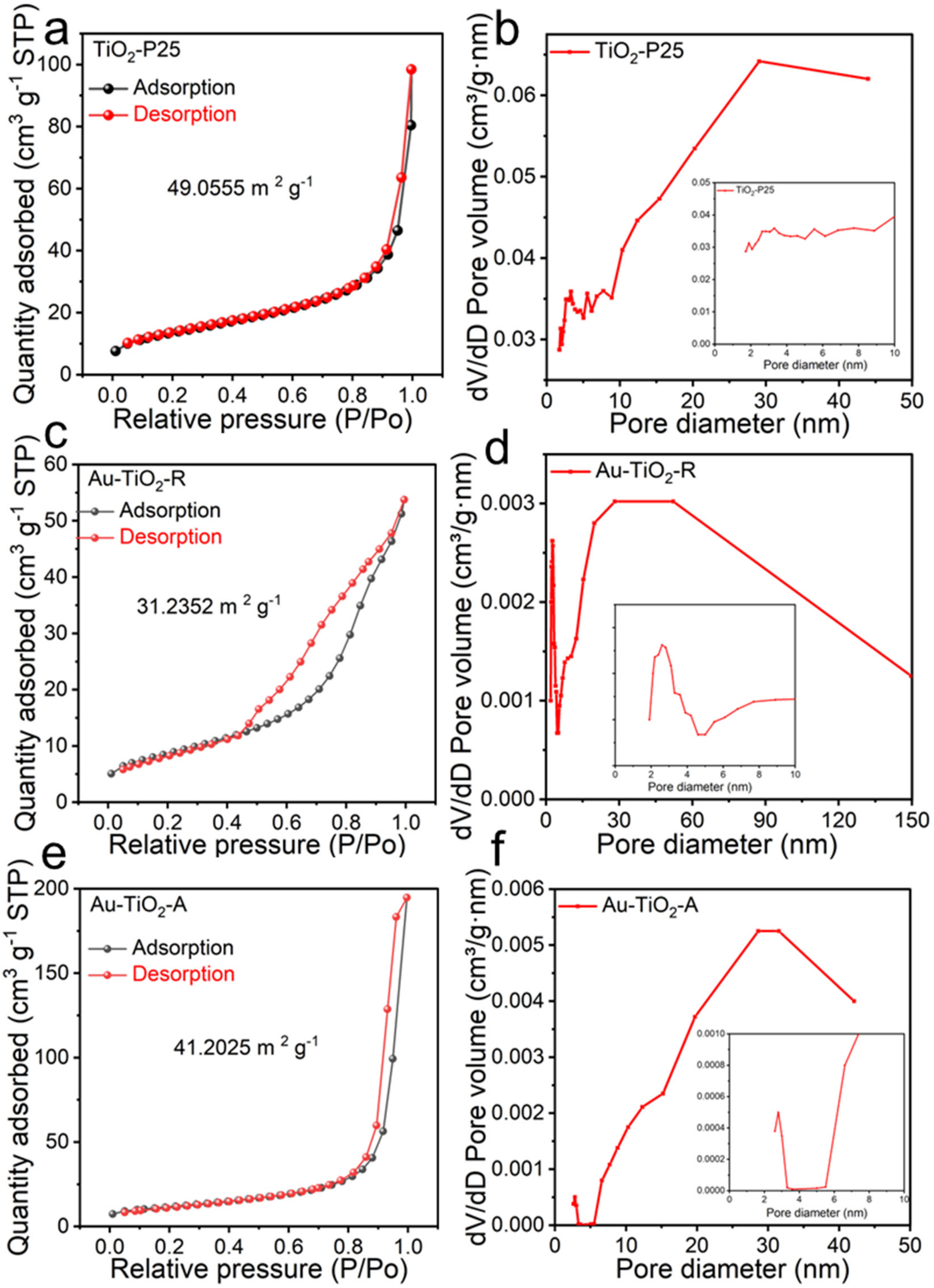

Figure S2. Nitrogen adsorption-desorption isothermal curves of materials and their pore sizes distributions (a-b) TiO_2-P25; (c-d) Au-TiO_2-R; (e-f) Au-TiO_2-A

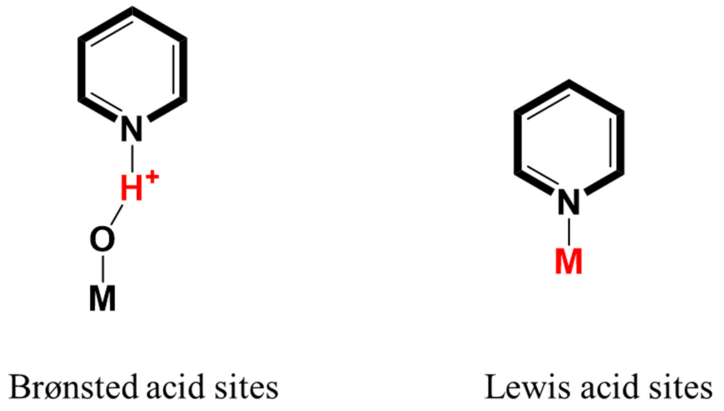

Figure S3. Diagram of pyridine adsorption on Brønsted acid sites and Lewis acid sites.

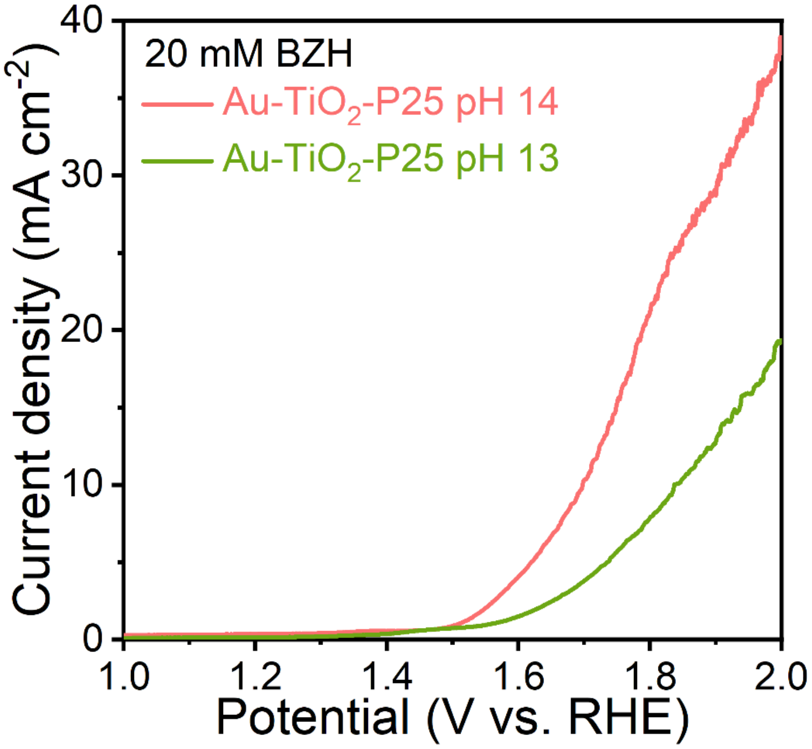

Figure S4. LSV curves of Au-TiO_2-P25 at a scan rate of 50 mV s^{-1} in 1 M KOH (pH 14) and 0.1 M KOH (pH 13).
